# A combination of mild-moderate hypoxemia and low compliance is highly prevalent in persistent ARDS: a retrospective study

**DOI:** 10.1186/s12931-023-02626-9

**Published:** 2024-01-03

**Authors:** Eleni Papoutsi, Ioannis Andrianopoulos, Vasiliki Mavrikaki, Maria Bolaki, Vagia Stamatopoulou, Eleni Toli, Georgios Papathanakos, Vasilios Koulouras, Eumorfia Kondili, Ilias I. Siempos, Katerina Vaporidi

**Affiliations:** 1 First Department of Critical Care Medicine and Pulmonary Services, Evangelismos Hospital, National and Kapodistrian University of Athens Medical School, Athens, Greece; 2 Department of Intensive Care Unit, University Hospital of Ioannina, Ioannina, Greece; 3 Department of Intensive Care, University Hospital of Heraklion, University of Crete School of Medicine, Voutes Campus, Office 8A4, Heraklion, Crete 70013 Greece; 4 Department of Medicine, Division of Pulmonary and Critical Care Medicine, Weill Cornell Medicine, New York, NY USA

**Keywords:** Acute respiratory failure, Acute respiratory distress syndrome, COVID-19, Prolonged mechanical ventilation

## Abstract

**Background:**

The Acute Respiratory Distress Syndrome (ARDS) is characterized by lung inflammation and edema, impairing both oxygenation and lung compliance. Recent studies reported a dissociation between oxygenation and compliance (severe hypoxemia with preserved compliance) in early ARDS and COVID-19-related-ARDS (CARDS). During the pandemic, in patients requiring prolonged mechanical ventilation, we observed the opposite combination (mild-moderate hypoxemia but significantly impaired compliance). The purpose of our study was to investigate the prevalence of this combination of mild-moderate hypoxemia and impaired compliance in persistent ARDS and CARDS.

**Methods:**

For this retrospective study, we used individual patient-level data from two independent cohorts of ARDS patients. The ARDSNet cohort included patients from four ARDS Network randomized controlled trials. The CARDS cohort included patients with ARDS due to COVID-19 hospitalized in two intensive care units in Greece. We used a threshold of 150 for PaO_2_/FiO_2_ and 30 ml/cmH_2_O for compliance, estimated the prevalence of each of the four combinations of oxygenation and compliance at baseline, and examined the change in its prevalence from baseline to day 21 in the ARDSNet and CARDS cohorts.

**Results:**

The ARDSNet cohort included 2909 patients and the CARDS cohort included 349 patients. The prevalence of the combination of mild-moderate hypoxemia and low compliance increased from baseline to day 21 both in the ARDSNet cohort (from 22.2 to 42.7%) and in the CARDS cohort (from 3.1 to 33.3%). Among surviving patients with low compliance, oxygenation improved over time. The 60-day mortality rate was higher for patients who had mild-moderate hypoxemia and low compliance on day 21 (28% and 56% in ARDSNet and CARDS), compared to those who had mild-moderate hypoxemia and high compliance (20% and 50%, respectively).

**Conclusions:**

Among patients with ARDS who require prolonged controlled mechanical ventilation, regardless of ARDS etiology, a dissociation between oxygenation and compliance characterized by mild-moderate hypoxemia but low compliance becomes increasingly prevalent. The findings of this study highlight the importance of monitoring mechanics in patients with persistent ARDS.

**Supplementary Information:**

The online version contains supplementary material available at 10.1186/s12931-023-02626-9.

## Introduction

Acute respiratory distress syndrome (ARDS) is defined as non-cardiogenic pulmonary edema causing hypoxemia [1]. Filling of the injured alveoli with protein-rich edema fluid [2], causes both an impairment in gas exchange and a reduction in lung compliance. Impairments in oxygenation and mechanics are thought to coincide and reflect the severity of ARDS [1]. However, it was observed that several patients with ARDS due to the coronavirus disease-19 (COVID-19) presented a dissociation between severe hypoxemia and relatively well-preserved compliance [3, 4]. A subsequent secondary analysis of the LUNG SAFE study revealed that approximately one in eight patients meeting the hypoxemia criterion of ARDS exhibited preserved compliance [5], confirming this dissociation between oxygenation and compliance in patients presenting with ARDS regardless of etiology. The above observations were limited to the first days after the onset of ARDS, i.e., at early stages.

The number of ARDS patients dramatically increased during the COVID-19 pandemic, as did patients who required prolonged mechanical ventilation [6]. During the pandemic, we observed that many patients after several days of mechanical ventilation exhibited an ‘opposite’ dissociation between oxygenation and compliance, characterized by mild-moderate hypoxemia but significantly reduced compliance.

Whether such a dissociation between oxygenation and compliance becomes prevalent in persistent ARDS regardless of etiology, or rather it is a particular feature of COVID-19 related ARDS is not known. This knowledge may be important given that a nonnegligible proportion of patients with ARDS require prolonged mechanical ventilation; i.e., they remain under ventilation more than 21 days after intubation [7]. Based on our observation, we endeavored to systematically examine the presence of this combination of mild-moderate hypoxemia and significantly impaired compliance, focusing specifically on patients with ARDS requiring prolonged mechanical ventilation.

## Methods

For this retrospective study, we used individual patient-level data from two independent cohorts of patients with ARDS.

The first cohort of our study (“ARDSNet” cohort) consisted of patients with ARDS enrolled in four prospective therapeutic clinical trials conducted by the ARDS Network, namely FACTT [8], ALTA [9], EDEN [10], and SAILS [11]. As previously [12, 13], we were granted access to data through the Biologic Specimen and Data Repository Information Coordinating Center (BioLINCC) of the National Heart, Lung, and Blood Institute (NHLBI). Because data would be received in de-identified form, the Institutional Review Board of Evangelismos Hospital waived the need for informed consent and approved the study.

The second cohort of our study (“CARDS” cohort) consisted of patients with ARDS related to COVID-19 admitted between October 2020 and January 2022 (i.e., when the alpha and delta Sars-Cov-2 variants were prominent) in two academic intensive care units (ICU) at tertiary hospitals in Crete and Ioannina, Greece. Part of data from those patients have been included in previously published observational studies [14, 15]. The Institutional Review Board at each participating study site (Ioannina: University Hospital of Ioannina, and Crete: University Hospital of Heraklion) approved of the data collection and waived the need for informed consent owing to the observational study design and the collection of de-identified data.

For both independent cohorts, we collected data on age, sex, Sequential Organ Failure Assessment (SOFA) score (both total SOFA score and non-respiratory SOFA score) at baseline, partial pressure of arterial oxygen to fraction of inspired oxygen ratio (PaO_2_:FiO_2_) and respiratory system compliance at baseline (day 0 or, if missing, day 1) and on days 7, 12 or 14 and 21. Day 0 was the day of enrollment for the ARDSNet cohort, and the day of intubation for the CARDS cohort. Data on day 12 were available from the ALTA, EDEN, and SAILS studies, while data on day 14 were available from the FACTT trial and the CARDS cohort. None of the patients were treated with extracorporeal oxygenation or carbon dioxide removal.

We used a threshold for low PaO_2_:FiO_2_ a value below 150, which has been used to identify patients with significant hypoxemia likely to benefit from prone position [16], and for low compliance a value below 30 mL/cmH_2_O, which has been shown to be associated with sustained high driving pressure and weaning failure [17, 18]. Accordingly, we considered four combinations of oxygenation and compliance in our study: mild-moderate hypoxemia/low compliance, mild-moderate hypoxemia/high compliance, severe hypoxemia/high compliance, and severe hypoxemia/low compliance.

We first analyzed all patients at baseline, and evaluated the prevalence of each of the four combinations. Data from all patients were included in this analysis (no exclusion criteria). In the ARDSNet cohort, we also conducted a sensitivity analysis by including only those patients who were randomized to the control group in the trials. We subsequently focused on patients who were on mechanical ventilation and had available data on oxygenation (as assessed by PaO_2_:FiO_2_) and respiratory system compliance on day 21. The primary endpoint of the study was the change in prevalence of the combination of mild-moderate hypoxemia/low compliance over time from baseline to day 21 among these patients. Secondary endpoints included: the difference in the prevalence of the combination of mild-moderate hypoxemia and low compliance between the ARDSNet cohort and the CARDS cohort; the trajectory of oxygenation among patients with either low or high compliance (using the abovementioned threshold of 30 mL/cmH_2_O) from baseline to day 21; the mortality at day 21 based on the oxygenation-compliance combination at baseline, and the 60-day mortality based on the oxygenation-compliance combination on day 21.

We presented continuous variables as medians with interquartile range (IQR) and compared them using the Mann-Whitney test (for 2 groups) or the Kruskal-Wallis test (for multiple groups). We presented categorical variables as percentages and compared them using the chi-squared or Fisher’s exact test, and corrected for multiple comparisons as appropriate. No formal sample size calculation prior to this purely observational study was performed. All p values were two-sided, and we considered statistical significance at an α level of 0.05. Statistical analyses were conducted using SPSS software version 28.0 (SPSS, Inc., Chicago, IL), and alluvial plots using R software version 4.2.1 (R Foundation for Statistical Computing).

## Results

### Characteristics of included patients in the two cohorts

**Fig. 1:**
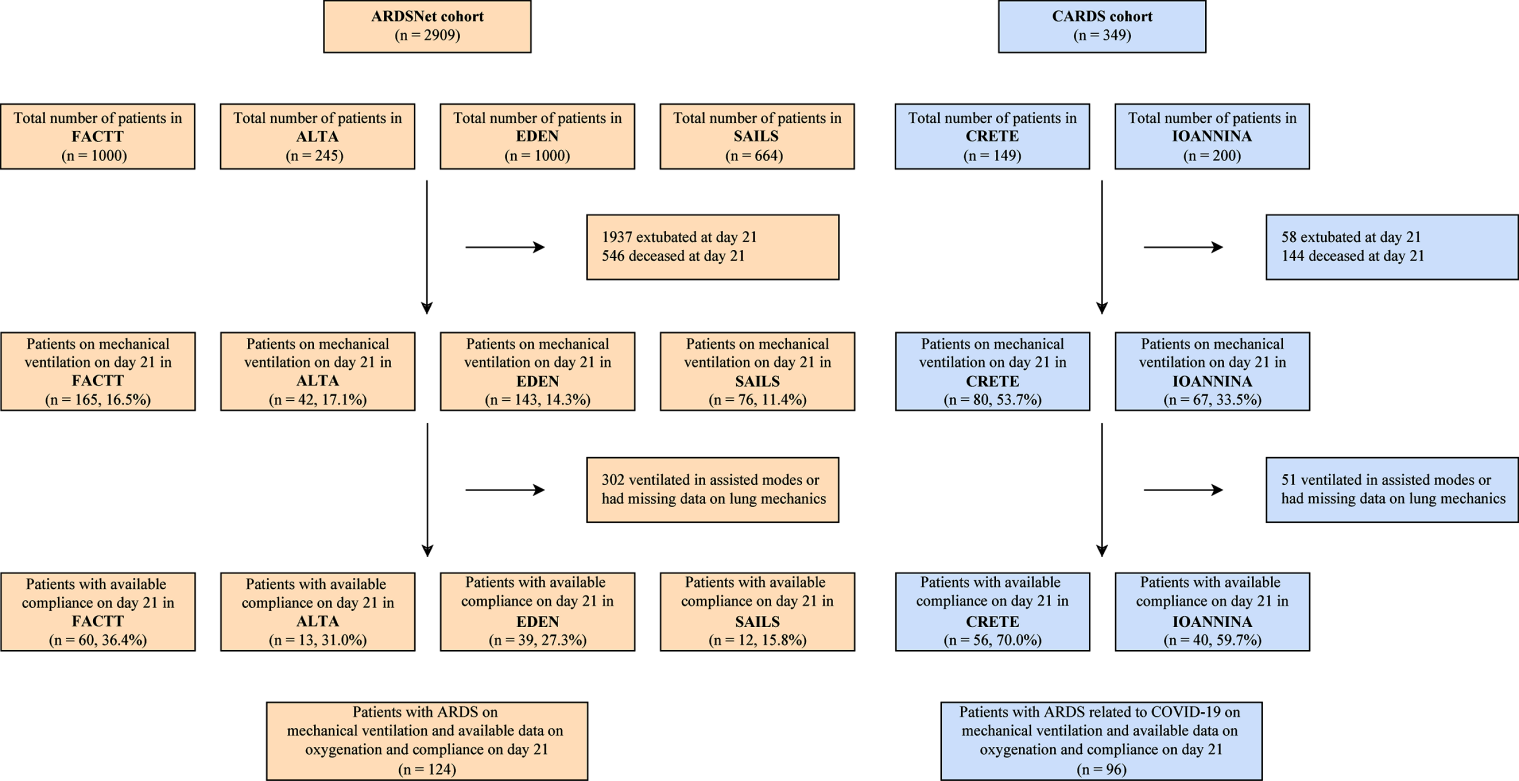
Patient flow diagram

Figure 1 and Supplemental Fig. 1 present the patient flow diagram for both cohorts (ARDSNet and CARDS). In the ARDSNet cohort, out of the 2909 patients with ARDS, the majority of patients (67%) were liberated from mechanical ventilation (“extubated” group) by day 21, 19% of patients had died (“deceased” group) by day 21, 10% remained on mechanical ventilation but did not have data on compliance, mostly because they were ventilated on assisted or partially assisted modes (“ventilated in assisted modes” group) by day 21, and 4% of patients were on mechanical ventilation and had available data on oxygenation and compliance (“ventilated in control modes” group) on day 21. In the CARDS cohort, out of the 349 patients, 17% were in the “extubated” group, 41% in the “deceased”, 15% in the “ventilated in assisted modes” and 28% were in the “ventilated in control modes” group.

Table 1 presents the baseline characteristics of patients categorized based on their status on day 21 (i.e., ventilated in control or assisted modes, extubated, or deceased) in the ARDSNet and CARDS cohorts. The combination of mild-moderate hypoxemia and low compliance at baseline was less common among patients ventilated in control modes compared to other groups in the ARDSNet cohort but not in the CARDS cohort.

**Table 1:** Baseline characteristics of patients according to status on day 21 of ARDSNet and CARDS cohort

	ARDSNet cohort				CARDS cohort			
	Ventilated in Control modes^1^ (n = 124)	Ventilated in Assisted modes^1^ (n = 302)	Extubated (n = 1937)	Deceased (n = 546)	Ventilated in Control modes^1^ (n = 96)	Ventilated in Assisted modes^1^ (n = 51)	Extubated (n = 58)	Deceased (n = 144)
Age	52.5 (41.0–65.8)	54.0 (42.0–65.0)	50.0 (39.0–60.0)*	58.0 (45.0–71.0)*	71.0 (61.0–75.8)	69.0 (61.0–74.0)	63.5 (53.0–75.0)**	72.0 (64.0–77.0)
Female sex n (%)	50 (40.3)	138 (45.7)	975 (50.3)	246 (45.1)	32 (33.3)	20 (39.2)	23 (39.7)	45 (31.5)
Baseline SOFA score	7 (5–9)	7 (5–9)	6 (4–8)^#^	8 (6–10)^#^	4 (4–6)	4 (4–8)	4 (4–6)^##^	5 (4–7)^##^
Non-respiratory SOFA	3 (1–6)	4 (2–6)^$^	3 (1–5)	5 (3–7)^$^	1 (0–3)	1 (0–4)	1 (0–2)	1 (0–3)
PaO_2_:FiO_2_ at baseline	103 (81–147)˚	146 (107–195)	150 (106–200)	127 (89–177)˚	120 (88–153)	131 (110–181)	118 (101–158)	101 (76–147)˚˚
Crs at baseline	27 (22–35)	28 (21–36)	30 (23–40)¨	28 (21–38)	36 (30–45)	40 (35–48)	38 (33–45)	32 (26–39)¨¨
MH-LoC at baseline^2^	13/108 (12.0)^•^	56/223 (25.1)	357/1527 (23.4)	76/405 (18.8)	2/94 (2.1)	0/43 (0.0)	2/55 (3.6)	6/128 (4.7)

Abbreviations: ARDS, acute respiratory distress syndrome; CARDS, COVID-19-related acute respiratory distress syndrome; n, number; SOFA, sequential organ failure assessment; PaO_2_:FiO_2_, partial pressure of arterial oxygen to fraction of inspired oxygen ratio; Crs, compliance; MH-LoC, mild-moderate hypoxemia and low compliance

Age, baseline SOFA, non-respiratory SOFA and lung mechanics at baseline are presented as median with interquartile range (IQR) and compared using the Kruskal-Wallis test. Sex is presented as percentage and compared using the chi-squared test. Pairwise comparisons were corrected using the Bonferroni method, and statistical significance was considered at an α level of 0.05

^1^The “ventilated in control modes” group consisted of patients who were on mechanical ventilation and had available data on oxygenation and compliance on day 21; the “ventilated in assisted modes” group consisted of patients who remained on mechanical ventilation but did not have data on compliance, mostly because they were ventilated on assisted or partially assisted modes by day 21; the “extubated” group consisted of patients who were liberated from mechanical ventilation by day 21; and the “deceased” group consisted of patients who died by day 21

^2^Due to missing data on respiratory system compliance at baseline, the total number of patients is less for this variable (shown as denominator)

*Deceased vs. all other groups p < 0.05, and extubated vs. ventilated in assisted modes p < 0.001; ^#^ Deceased vs. all other groups, and extubated vs. ventilated in control, assisted modes p < 0.05; ^$^ Deceased vs. extubated, ventilated in control modes, and ventilated in assisted modes vs. extubated p < 0.001; ˚ Deceased vs. all other groups p < 0.05, and ventilated in control modes vs. ventilated in assisted modes, extubated p < 0.001; ¨ Extubated vs. deceased, ventilated in assisted modes p < 0.05; ^•^Ventilated in control modes vs. ventilated in assisted modes, extubated p < 0.05

**Extubated vs. ventilated in control modes, deceased p < 0.05; ^##^Deceased vs. extubated p < 0.05; ˚˚Deceased vs. ventilated in assisted modes p < 0.01; ¨¨ Deceased vs. all other groups p < 0.01

Supplemental Table 1 presents the comparison of baseline characteristics of patients included in the ARDSNet versus the CARDS cohort. Regardless of their status on day 21, patients in the ARDSNet cohort were younger, had higher total SOFA score, higher non-respiratory SOFA score and lower respiratory system compliance at baseline compared to patients in the CARDS cohort. The combination of mild-moderate hypoxemia but low compliance at baseline was more common in the ARDSNet cohort than in the CARDS cohort.

### Trajectory of oxygenation and compliance from baseline to day 21

In the ARDSNet cohort, the combination of mild-moderate hypoxemia but low compliance increased among patients ventilated in control mode from a prevalence of 22.2% at baseline to 42.7% on day 21 (p < 0.001). A sensitivity analysis including only patients who were randomized to the control group showed similar results, specifically the combination of mild-moderate hypoxemia but low compliance increased from a prevalence of 23.1% at baseline to 47.8% on day 21 (p < 0.001). Likewise, in the CARDS cohort, the combination of mild-moderate hypoxemia but low compliance increased from a prevalence of 3.1% at baseline to 33.3% on day 21 (p < 0.001). The prevalence of the combination of mild-moderate hypoxemia but low compliance on day 21 was similar in both the ARDSNet and CARDS cohorts (p = 0.155).

**Fig. 2:**
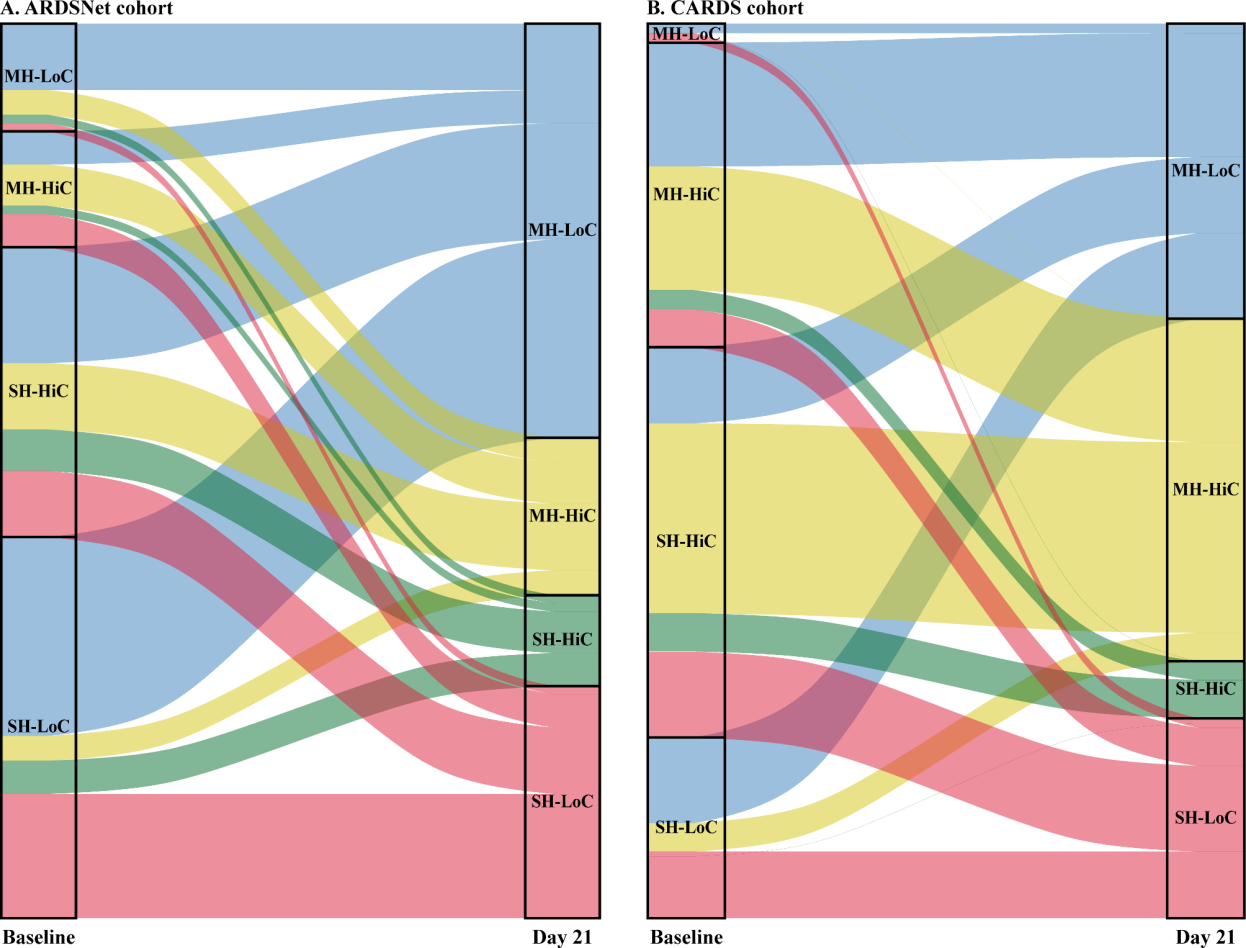
Alluvial plot of changes in prevalence of different combinations of oxygenation and compliance over time from baseline to day 21, among patients who remained on controlled ventilation and had available data on oxygenation and compliance, in (**A**) the ARDSNet cohort, and (**B**) the CARDS cohort. Each block represents a different combination, and the height of a block represents the number of patients in this combination. Combinations at baseline are illustrated with different colors. Each stream field between two blocks represents the change in the number of patients in the respective combinations from baseline to day 21, and the height of a stream field is proportionate to this number of patients. A total of 108 and 94 patients with available data on oxygenation and compliance on both baseline and day 21 are included in the alluvial plot of the ARDSNet and CARDS cohort, respectively. MH-HiC: mild-moderate hypoxemia/high compliance; MH-LoC: mild-moderate hypoxemia/low compliance; SH-HiC: severe hypoxemia/high compliance; and SH-LoC: severe hypoxemia/low compliance

After limiting our analysis to only those patients ventilated in control modes on day 21, the combination of mild-moderate hypoxemia but low compliance increased from a prevalence of 7.4% (12.0% and 2.1% in the ARDSNet and CARDS cohort, respectively) at baseline to 38.6% (42.7% and 33.3% in the ARDSNet and CARDS cohort, respectively) on day 21 (p < 0.001 for all comparisons). Alluvial plots to depict the change in prevalence of the different combinations over time from baseline to day 21 are shown in Fig. 2. In the sensitivity analysis including only patients from the ARDSNet cohort with pneumonia as the primary risk factor of ARDS (Supplemental Table 2), the combination of mild-moderate hypoxemia but low compliance increased from a prevalence of 5.4% at baseline to 34.6% on day 21 (p < 0.001).

**Fig. 3:**
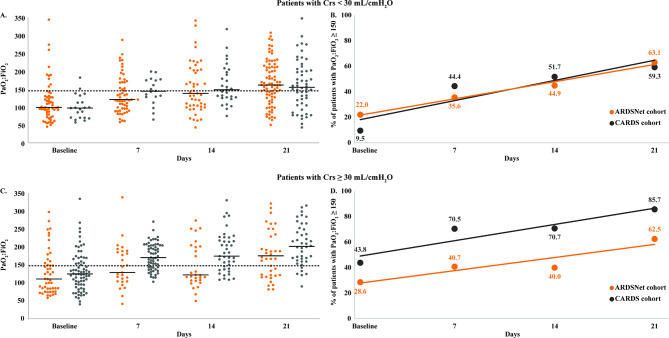
(**A**) Trajectory of oxygenation, as assessed by the partial pressure of arterial oxygen to fraction of inspired oxygen ratio (PaO_2_:FiO_2_), (line at median), and (**B**) percent of patients with mild-moderate hypoxemia, among patients with low compliance at baseline and on days 7, 14 and 21 in the two independent cohorts (ARDSNet and CARDS). (**C**) Trajectory of oxygenation, as assessed by the partial pressure of arterial oxygen to fraction of inspired oxygen ratio (PaO_2_:FiO_2_), and (**D**) percent of patients with mild-moderate hypoxemia, among patients with high compliance at baseline and on days 7, 14 and 21 in the two independent cohorts

Among patients who had low compliance, in whom the underlying pathophysiology, edema or atelectasis causing the impairment in mechanics, would be expected to affect oxygenation, oxygenation appeared to improve over time (Fig. 3A-B). This was also the case for patients who had high compliance (Fig. 3C-D).

### Outcome

Supplemental Table 3 presents data on outcomes (i.e., extubated or dead) on day 21 of patients categorized by their oxygenation-compliance combination at baseline in both cohorts. The corresponding Kaplan-Meier curves are presented as Supplemental Fig. 2.

Supplemental Table 4 presents data on 60-day mortality of patients categorized by their oxygenation-compliance combination on day 21 in both cohorts. In the ARDSNet cohort, 60-day mortality was 28% in the mild-moderate hypoxemia/low compliance and 20% in the mild-moderate hypoxemia/high compliance combination, 67% in the severe hypoxemia/high compliance combination, and 32% in the severe hypoxemia/low compliance combination. In the CARDS cohort, 60-day mortality was 56% and 50% in the mild-moderate hypoxemia/low compliance and the mild-moderate hypoxemia/high compliance groups, and 82–83% in the severe hypoxemia with low and high compliance, respectively.

## Discussion

The results of this study highlight the dissociation between oxygenation and compliance during the late stages of ARDS, as a combination of mild-moderate hypoxemia but severely impaired compliance, becomes increasingly prevalent over time, regardless of ARDS etiology.

The dissociation between oxygenation and compliance in ARDS has recently gained attention [4]. Impairment of both oxygenation and lung compliance typically characterizes ARDS, and is the result of lung inflammation, impaired hypoxic pulmonary vasoconstriction and alveolar flooding [19–21]. The recently described dissociation between oxygenation and compliance in early ARDS patients [3, 4] is attributed to increased shunt due to vasculopathy, and impaired hypoxic pulmonary vasoconstriction [4, 5, 22].

To our knowledge, this is the first systematic examination of an “opposite” dissociation of oxygenation and compliance, observed in persistent ARDS, and characterized by mild-moderate hypoxemia but severely impaired compliance. This dissociation was indicated, but not systematically examined by previous studies [23, 24], which were mostly limited to the first week from the onset of the syndrome [25]. Rather, the present analysis took advantage of longitudinal data on oxygenation-compliance for three weeks from the onset of ARDS, for both traditional ARDS and COVID-19-related ARDS. Specifically, our analysis showed that, although the combination of mild-moderate hypoxemia but low compliance was not as rare in early ARDS due to etiologies other than COVID-19 compared to COVID-19 (Supplemental Table 1), the combination became prominent among patients with persistent ARDS requiring controlled ventilation, regardless of etiology.

It may be worth attempting an explanation why this combination of mild-moderate hypoxemia and low compliance becomes increasingly prevalent over the course of ARDS among patients who remain under controlled mechanical ventilation. In persistent ARDS, the resolution of pulmonary inflammation may lead to restoration of hypoxic pulmonary vasoconstriction, which in turn may optimize the distribution of perfusion and thus attenuate hypoxemia [20, 26]. In spite of the attenuation of hypoxemia, compliance may be reduced due to the progressive development of some degree of fibrosis, known to occur in persistent ARDS [27]. Therefore, concurrent improvement in ventilation-perfusion matching and development of fibrosis over time may underlie the pathophysiology of improved oxygenation despite severely impaired compliance in persistent ARDS. The impaired compliance is likely the reason these patients could not be liberated from controlled ventilation, thus resulting in the increased prevalence of the combination of low compliance and mild-moderate hypoxemia among patients requiring prolonged controlled ventilation.

Recognizing this dissociation among patients with persistent ARDS may have important clinical implications. Hypoxemia dominates all diagnostic and management algorithms of ARDS, such as the Berlin definition [1], indication for prone position [16] or ECMO [28], and for a good reason: it is a cardinal symptom of ARDS at presentation, and lethal if not managed. The impairment in lung compliance is caused by the same underlying pathology, and commonly correlates with the severity of hypoxemia. As a result, resolution of hypoxemia over time is considered an adequate indicator of resolution of ARDS, and included in weaning protocols [8–11, 29], while improvement in lung mechanics is not. Indeed, during the first couple of weeks of ARDS, in the majority of patients who improve, both oxygenation and compliance improve. However, as highlighted in this analysis, in a significant fraction of patients requiring prolonged controlled mechanical ventilation, the improvement of hypoxemia is dissociated from that of lung compliance at late stages of ARDS. Weaning attempts when lung compliance is severely impaired might fail and place patients at risk of self-inflicted lung injury [30–32]. Indeed, strong inspiratory efforts may promote lung injury in ARDS, and sustained high driving pressures have been shown to occur during assisted ventilation only in patients with low compliance [17, 33]. Therefore, acknowledging this common dissociation between oxygenation and compliance in persistent ARDS, rather than relying solely on improvement of oxygenation to characterize a patient’s condition as improved, may have implications for patient management. For example, in patients with persistent ARDS, attempts for assisted ventilation could be complemented by close monitoring of effort and driving pressure, to minimize the risk of ventilator or self-inflicted lung injury [34, 35]. Studies on weaning focused on this specific group of patients are lacking and urgently needed.

The findings of this study should be interpreted in the context of the limitations imposed by its retrospective nature. First, although the ARDSNet cohort consisted of patients enrolled in multiple ICUs from one continent (North America), the CARDS cohort consisted of patients enrolled in only two ICUs from one European country. Second, the sample size is limited by missing data on compliance from patients who remained on mechanical ventilation on day 21, mainly because mechanics were not monitored on assisted modes. However, an exploratory analysis of CARDS patients indicated that both compliance and oxygenation had improved prior to assisted ventilation (data not shown). Third, to facilitate the analysis, we had to use thresholds for oxygenation and compliance, acknowledging the inherent limitations of using thresholds to create classes in the continuum of a disease spectrum. We also had to use the PaO_2_:FiO_2_ ratio at different levels of FiO_2_ as an index of oxygenation, despite its nonlinear behavior, as this is what is currently used in clinical practice. Finally, we cannot provide information on the underlying cause of impaired compliance, to what extent it contributed to the need for prolonged ventilation, or its management in the surviving patients.

## Conclusions

In conclusion, in persistent ARDS regardless of the etiology, a combination of mild-moderate hypoxemia but low compliance becomes prevalent among patients who remain under controlled ventilation. Acknowledging that improvement in oxygenation is not always associated with restoration of lung integrity in persistent ARDS, adds to the increasingly recognized importance of monitoring lung mechanics in mechanically ventilated patients, throughout the course of ARDS in order to provide protective ventilation.

### Electronic supplementary material

Below is the link to the electronic supplementary material.

**Supplementary Material 1:** **Supplemental Table 1.** Baseline characteristics of patients according to status on day 21. **Supplemental Table 2.** Baseline characteristics and lung mechanics of the patients with pneumonia as primary risk factor for ARDS. **Supplemental Table 3.** Outcomes on day 21 of patients categorized by their oxygenation-compliance combination at baseline. **Supplemental Table 4.** Data on 60-day mortality of patients categorized by their oxygenation-compliance combination on day 21. **Supplemental Figure 1.** Detailed patient flow diagram for both study cohorts. **Supplemental Figure 2.** Kaplan-Meier curves of mortality up to day 21 of patients categorized by their oxygenation-compliance combination at baseline

## Data Availability

Data of patients from the ARDSNet cohort are available through the Biologic Specimen and Data Repository Information Coordinating Center of the National Heart, Lung, and Blood Institute (https://biolincc.nhlbi.nih.gov/home/). Data of patients from the CARDS cohort are available from the corresponding author on reasonable request.

## List of abbreviations

ARDS: Acute respiratory distress syndrome
COVID-19: Coronavirus disease 2019
CARDS: COVID-19-related ARDS
ICU: Intensive care unit
BioLINCC: Biologic Specimen and Data Repository Information Coordinating Center
NHLBI: National Heart, Lung, and Blood Institute
PaO_2_:FiO_2_: Partial pressure of arterial oxygen to fraction of inspired oxygen ratio
Crs: Respiratory system compliance
SOFA: Sequential Organ Failure Assessment
VT: Tidal volume
PEEP: Positive end expiratory pressure
Ppl: Plateau pressure
MH-LoC: mild-moderate hypoxemia/low compliance
MH-HiC: mild-moderate hypoxemia /high compliance
SH-HiC: severe hypoxemia /high compliance
SH-LoC: severe hypoxemia /low compliance

## References

[1] Ranieri VM, Rubenfeld GD, Thompson BT, Ferguson ND, Caldwell E, Fan E, Camporota L, Slutsky AS (2012). Acute respiratory distress syndrome: the Berlin definition. JAMA.

[2] Ware LB, Matthay MA (2000). The acute respiratory distress syndrome. N Engl J Med.

[3] Gattinoni L, Coppola S, Cressoni M, Busana M, Rossi S, Chiumello D (2020). COVID-19 does not lead to a typical Acute Respiratory Distress Syndrome. Am J Respir Crit Care Med.

[4] Gattinoni L, Chiumello D, Caironi P, Busana M, Romitti F, Brazzi L, Camporota L. COVID-19 Pneumonia: different respiratory treatments for different phenotypes? Intensive care Med. United States; 2020. pp. 1099–102. 10.1007/s00134-020-06033-2 PMC7154064 32291463

[5] Panwar R, Madotto F, Laffey JG, van Haren FMP (2020). Compliance phenotypes in early acute respiratory distress syndrome before the COVID-19 pandemic. Am J Respir Crit Care Med.

[6] Bain W, Yang H, Shah FA, Suber T, Drohan C, Al-Yousif N, DeSensi RS, Bensen N, Schaefer C, Rosborough BR, Somasundaram A, Workman CJ, Lampenfeld C, Cillo AR, Cardello C, Shan F, Bruno TC, Vignali DAA, Ray P, Ray A, Zhang Y, Lee JS, Methé B, McVerry BJ, Morris A, Kitsios GD (2021). COVID-19 versus Non-COVID-19 Acute Respiratory Distress Syndrome: comparison of demographics, physiologic parameters, inflammatory biomarkers, and clinical outcomes. Ann Am Thorac Soc.

[7] MacIntyre NR, Epstein SK, Carson S, Scheinhorn D, Christopher K, Muldoon S. Management of patients requiring prolonged mechanical ventilation: report of a NAMDRC consensus conference. *Chest* 2005; 128: 3937–3954. 10.1378/chest.128.6.3937 16354866

[8] Wiedemann HP, Wheeler AP, Bernard GR, Thompson BT, Hayden D, deBoisblanc B, Connors AF, Hite RD, Harabin AL (2006). Comparison of two fluid-management strategies in acute lung injury. N Engl J Med.

[9] Matthay MA, Brower RG, Carson S, Douglas IS, Eisner M, Hite D, Holets S, Kallet RH, Liu KD, MacIntyre N, Moss M, Schoenfeld D, Steingrub J, Thompson BT (2011). Randomized, placebo-controlled clinical trial of an aerosolized β_2_-agonist for treatment of acute lung injury. Am J Respir Crit Care Med.

[10] Rice TW, Wheeler AP, Thompson BT, Steingrub J, Hite RD, Moss M, Morris A, Dong N, Rock P (2012). Initial trophic vs full enteral feeding in patients with acute lung injury: the EDEN randomized trial. JAMA.

[11] Truwit JD, Bernard GR, Steingrub J, Matthay MA, Liu KD, Albertson TE, Brower RG, Shanholtz C, Rock P, Douglas IS, deBoisblanc BP, Hough CL, Hite RD, Thompson BT (2014). Rosuvastatin for sepsis-associated acute respiratory distress syndrome. N Engl J Med.

[12] Papoutsi E, Routsi C, Kotanidou A, Vaporidi K, Siempos II (2021). Association between driving pressure and mortality may depend on timing since onset of acute respiratory distress syndrome. Intensive Care Med.

[13] Andrianopoulos I, Giannakoulis VG, Papoutsi E, Papathanakos G, Koulouras V, Thompson BT, Siempos II. Prolonged mechanical ventilation in acute respiratory distress syndrome. *Shock* 2023; [In press]. 10.1097/SHK.0000000000002248 38010051

[14] Gavrielatou E, Vaporidi K, Tsolaki V, Tserlikakis N, Zakynthinos GE, Papoutsi E, Maragkuti A, Mantelou AG, Karayiannis D, Mastora Z, Georgopoulos D, Zakynthinos E, Routsi C, Zakynthinos SG, Schenck EJ, Kotanidou A, Siempos II (2022). Rapidly improving acute respiratory distress syndrome in COVID-19: a multi-centre observational study. Respir Res.

[15] Grapsa E, Adamos G, Andrianopoulos I, Tsolaki V, Giannakoulis VG, Karavidas N, Giannopoulou V, Sarri K, Mizi E, Gavrielatou E, Papathanakos G, Mantzarlis KD, Mastora Z, Magira E, Koulouras V, Kotanidou A, Siempos II (2022). Association between Vaccination Status and Mortality among intubated patients with COVID-19-Related Acute Respiratory Distress Syndrome. JAMA Netw Open.

[16] Guérin C, Reignier J, Richard JC, Beuret P, Gacouin A, Boulain T, Mercier E, Badet M, Mercat A, Baudin O, Clavel M, Chatellier D, Jaber S, Rosselli S, Mancebo J, Sirodot M, Hilbert G, Bengler C, Richecoeur J, Gainnier M, Bayle F, Bourdin G, Leray V, Girard R, Baboi L, Ayzac L (2013). Prone positioning in severe acute respiratory distress syndrome. N Engl J Med.

[17] Vaporidi K, Psarologakis C, Proklou A, Pediaditis E, Akoumianaki E, Koutsiana E, Chytas A, Chouvarda I, Kondili E, Georgopoulos D (2019). Driving pressure during proportional assist ventilation: an observational study. Ann Intensive Care.

[18] Yang KL, Tobin MJ (1991). A prospective study of indexes predicting the outcome of trials of weaning from mechanical ventilation. N Engl J Med.

[19] Matthay MA, Zemans RL, Zimmerman GA, Arabi YM, Beitler JR, Mercat A, Herridge M, Randolph AG, Calfee CS (2019). Acute respiratory distress syndrome. Nat Rev Dis Primers.

[20] Gierhardt M, Pak O, Walmrath D, Seeger W, Grimminger F, Ghofrani HA, Weissmann N, Hecker M, Sommer N. Impairment of hypoxic pulmonary vasoconstriction in acute respiratory distress syndrome. Eur Respir Rev 2021; 30. 10.1183/16000617.0059-2021 PMC9489056 34526314

[21] Bos LDJ, Ware LB (2022). Acute respiratory distress syndrome: causes, pathophysiology, and phenotypes. Lancet.

[22] Habashi NM, Camporota L, Gatto LA, Nieman G (2021). Functional pathophysiology of SARS-CoV-2-induced acute lung injury and clinical implications. J Appl Physiol (1985).

[23] Meduri GU, Chinn AJ, Leeper KV, Wunderink RG, Tolley E, Winer-Muram HT, Khare V, Eltorky M (1994). Corticosteroid rescue treatment of Progressive fibroproliferation in late ARDS. Patterns of response and predictors of outcome. Chest.

[24] Raurich JM, Ferreruela M, Llompart-Pou JA, Vilar M, Colomar A, Ayestarán I, Pérez-Bárcena J, Ibáñez J (2012). Potential effects of corticosteroids on physiological dead-space fraction in acute respiratory distress syndrome. Respir Care.

[25] Li Bassi G, Suen JY, Dalton HJ, White N, Shrapnel S, Fanning JP, Liquet B, Hinton S, Vuorinen A, Booth G, Millar JE, Forsyth S, Panigada M, Laffey J, Brodie D, Fan E, Torres A, Chiumello D, Corley A, Elhazmi A, Hodgson C, Ichiba S, Luna C, Murthy S, Nichol A, Ng PY, Ogino M, Pesenti A, Trieu HT, Fraser JF (2021). An appraisal of respiratory system compliance in mechanically ventilated covid-19 patients. Crit Care.

[26] Dunham-Snary KJ, Wu D, Sykes EA, Thakrar A, Parlow LRG, Mewburn JD, Parlow JL, Archer SL (2017). Hypoxic pulmonary vasoconstriction: from Molecular mechanisms to Medicine. Chest.

[27] Hudson LD, Hough CL (2006). Therapy for late-phase acute respiratory distress syndrome. Clin Chest Med.

[28] Combes A, Hajage D, Capellier G, Demoule A, Lavoué S, Guervilly C, Da Silva D, Zafrani L, Tirot P, Veber B, Maury E, Levy B, Cohen Y, Richard C, Kalfon P, Bouadma L, Mehdaoui H, Beduneau G, Lebreton G, Brochard L, Ferguson ND, Fan E, Slutsky AS, Brodie D, Mercat A (2018). Extracorporeal membrane oxygenation for severe Acute Respiratory Distress Syndrome. N Engl J Med.

[29] Pham T, Heunks L, Bellani G, Madotto F, Aragao I, Beduneau G, Goligher EC, Grasselli G, Laake JH, Mancebo J, Peñuelas O, Piquilloud L, Pesenti A, Wunsch H, van Haren F, Brochard L, Laffey JG. Weaning from mechanical ventilation in intensive care units across 50 countries (WEAN SAFE): a multicentre, prospective, observational cohort study. Lancet Respir Med 2023. 10.1016/S2213-2600(22)00449-0 36693401

[30] Yoshida T, Fujino Y, Amato MB, Kavanagh BP (2017). Fifty years of Research in ARDS. Spontaneous breathing during mechanical ventilation. Risks, mechanisms, and management. Am J Respir Crit Care Med.

[31] Mauri T, Cambiaghi B, Spinelli E, Langer T, Grasselli G (2017). Spontaneous breathing: a double-edged sword to handle with care. Ann Transl Med.

[32] Heunks LM, van der Hoeven JG (2010). Clinical review: the ABC of weaning failure–a structured approach. Crit Care.

[33] Morais CCA, Koyama Y, Yoshida T, Plens GM, Gomes S, Lima CAS, Ramos OPS, Pereira SM, Kawaguchi N, Yamamoto H, Uchiyama A, Borges JB, Vidal Melo MF, Tucci MR, Amato MBP, Kavanagh BP, Costa ELV, Fujino Y (2018). High positive end-expiratory pressure renders spontaneous effort Noninjurious. Am J Respir Crit Care Med.

[34] Goligher EC, Dres M, Patel BK, Sahetya SK, Beitler JR, Telias I, Yoshida T, Vaporidi K, Grieco DL, Schepens T, Grasselli G, Spadaro S, Dianti J, Amato M, Bellani G, Demoule A, Fan E, Ferguson ND, Georgopoulos D, Guérin C, Khemani RG, Laghi F, Mercat A, Mojoli F, Ottenheijm CAC, Jaber S, Heunks L, Mancebo J, Mauri T, Pesenti A, Brochard L (2020). Lung- and diaphragm-protective ventilation. Am J Respir Crit Care Med.

[35] Yoshida T, Fujino Y (2021). Monitoring the patient for a safe-assisted ventilation. Curr Opin Crit Care.

