# Supplementary material for: A combination of mild-moderate hypoxemia and low compliance is highly prevalent in persistent ARDS: a retrospective study
Source: Respir Res. 2024 Jan 3;25:1. doi: 10.1186/s12931-023-02626-9 (PMC10765810; doi:10.1186/s12931-023-02626-9)

**Online Data Supplement**

**A combination of mild-moderate hypoxemia and low compliance is highly prevalent in persistent ARDS: a retrospective study**

**Supplemental Table 1.**  Baseline characteristics of patients according to status on day 21.

**Supplemental Table 2.** Baseline characteristics and lung mechanics of the patients with pneumonia as primary risk factor for ARDS.

**Supplemental Table 3.** Outcomes on day 21 of patients categorized by their oxygenation-compliance combination at baseline.

**Supplemental Table 4.** Data on 60-day mortality of patients categorized by their oxygenation-compliance combination on day 21.

**Supplemental Figure 1.** Detailed patient flow diagram for both study cohorts.

**Supplemental Figure 2.** Kaplan-Meier curves of mortality up to day 21 of patients categorized by their oxygenation-compliance combination at baseline.

| **Supplemental Table 1.**  **Baseline characteristics of patients according to status on day 21.** | | | | | | | | | | | | |
| --- | --- | --- | --- | --- | --- | --- | --- | --- | --- | --- | --- | --- |
|  | **Ventilated in Control modes^1^** | | | **Ventilated in Assisted modes^1^** | | | **Extubated** | | | **Deceased** | | |
|  | **ARDSNet (n=124)** | **CARDS  (n=96)** | **p value** | **ARDSNet (n=302)** | **CARDS  (n=51)** | **p value** | **ARDSNet (n=1937)** | **CARDS  (n=58)** | **p value** | **ARDSNet (n=546)** | **CARDS  (n=144)** | **p  value** |
| Age | 52.5  (41.0 - 65.8) | 71.0  (61.0 - 75.8) | <0.001 | 54.0  (42.0 - 65.0) | 69.0  (61.0 - 74.0) | <0.001 | 50.0  (39.0 - 60.0) | 63.5  (53.0 - 75.0) | <0.001 | 58.0  (45.0 - 71.0) | 72.0  (64.0 - 77.0) | <0.001 |
| Female sex %  (n) | 50  (40.3) | 32  (33.3) | 0.288 | 138  (45.7) | 20  (39.2) | 0.389 | 975  (50.3) | 23  (39.7) | 0.109 | 246  (45.1) | 45  (31.5) | 0.003 |
| Total SOFA score | 7  (5 - 9) | 4  (4 - 6) | <0.001 | 7  (5 - 9) | 4  (4 - 8) | <0.001 | 6  (4 - 8) | 4  (4 - 6) | <0.001 | 8  (6 - 10) | 5  (4 -7) | <0.001 |
| Non-respiratory SOFA score | 3  (1 - 6) | 1  (0 - 3) | <0.001 | 4  (2 - 6) | 1  (0 - 4) | <0.001 | 3  (1 - 5) | 1  (0 - 2) | <0.001 | 5  (3 - 7) | 1  (0 - 3) | <0.001 |
| PaO_2_:FiO_2_ at baseline | 103  (81 - 147) | 120  (88 - 153) | 0.266 | 146  (107 - 195) | 131 (110 - 181) | 0.289 | 150  (106 - 200) | 118 (101 - 158) | 0.003 | 127  (89 - 177) | 101 (76 - 147) | <0.001 |
| Crs at baseline^2^ | 27  (22 - 35) | 36 (30 - 45) | <0.001 | 28  (21 - 36) | 40  (35 - 48) | <0.001 | 30  (23 - 40) | 38 (33 - 45) | <0.001 | 28 (21 - 38) | 32  (26 - 39) | 0.001 |
| MH-LoC at baseline^2^ | 13/108  (12.0) | 2/94  (2.1) | 0.007 | 56/223  (25.1) | 0/43  (0.0) | <0.001 | 357/1527  (23.4) | 2/55  (3.6) | <0.001 | 76/405  (18.8) | 6/128 (4.7) | <0.001 |

*Abbreviations*: ARDS, acute respiratory distress syndrome; CARDS, COVID-19-related ARDS; n, number; SOFA, sequential organ failure assessment; PaO_2_:FiO_2_, partial pressure of arterial oxygen to fraction of inspired oxygen ratio; Crs, compliance; MH-LoC, mild-moderate hypoxemia and low compliance.

^1^Study population consisted of patients who were ventilated in control modes, and had available data on oxygenation and Crs on day 21. Most of the patients on mechanical ventilation without available data on mechanics were on assisted modes (details on the modes of ventilation are not available for patients in the FACTT ARDSNet trial). ^2^Due to missing data on respiratory system compliance at baseline, the total number of patients is less for this variable (shown as denominator).

| **Supplemental Table 2.** **Baseline characteristics and lung mechanics of patients with pneumonia as primary risk factor for ARDS.** | | | |
| --- | --- | --- | --- |
|  | ARDSNet cohort  (n=66) | CARDS cohort (n=96) | p value |
| Age | 51.5  (42.0 - 63.3) | 71.0 (61.0 - 75.8) | <0.001 |
| Female sex % (n) | 31 (47.0) | 32 (33.3) | 0.080 |
| Baseline SOFA score | 6  (5 - 8) | 4.0 (4.0 - 6.0) | <0.001 |
| Baseline non-respiratory SOFA score | 3  (1 - 5) | 1.0 (0.0 - 3.0) | <0.001 |
| PaO_2_:FiO_2_ | 99  (76 - 130) | 120 (88 – 153) | 0.058 |
| Crs | 27  (21 - 35) | 36 (30 - 45) | <0.001 |
| MH-LoC | 6/55 (10.9) | 2/94 (2.1) | 0.052 |
| 60-day mortality | 22 (33.3) | 59 (61.5) | <0.001 |

*Abbreviations:* ARDS, acute respiratory distress syndrome; CARDS, COVID-19-related ARDS; n, number; SOFA, sequential organ failure assessment; PaO_2_:FiO_2_, partial pressure of arterial oxygen to fraction of inspired oxygen ratio; Crs, compliance; MH-LoC, mild-moderate hypoxemia and low compliance.

| **Supplemental Table 3. Outcomes on day 21 of patients categorized by their oxygenation-compliance combination at baseline.** | | | | | | | | | | | | | |
| --- | --- | --- | --- | --- | --- | --- | --- | --- | --- | --- | --- | --- | --- |
|  | **ARDSNet cohort (n=2909)** | | | | | | **CARDS cohort (n=349)** | | | | | | |
|  | MH-LoC (n = 502) | MH-HiC (n = 548) | SH-HiC (n = 561) | SH-LoC (n = 652) | Missing (n = 646) | p value | MH-LoC  (n = 10) | MH-HiC (n = 96) | SH-HiC (n = 140) | SH-LoC  (n = 74) | Missing (n = 29) | p value |  |
| Extubated | 359 (71.5) | 421 (76.8) | 367 (65.4) | 382 (58.6) | 411 (63.6) | <0.001 | 2 (20.0) | 17 (17.7) | 31 (22.1) | 5 (6.8) | 3 (10.3) | 0.055 |  |
| 21-day mortality | 76 (15.1) | 69 (12.6) | 110 (19.6) | 153 (23.5) | 141 (21.8) | <0.001 | 6 (60.0) | 32 (33.3) | 46 (32.9) | 44 (59.5) | 16 (55.2) | <0.001 |  |

*Abbreviations:* ARDS, acute respiratory distress syndrome; CARDS, COVID-19-related ARDS; MH-LoC, mild-moderate hypoxemia and low compliance; MH-HiC, mild-moderate hypoxemia and high compliance; SH-HiC, severe hypoxemia and high compliance; SH-LoC, severe hypoxemia and low compliance; n, number.

Baseline was day 0 or, if missing, day 1.

The “Missing” category includes patients that did not have compliance measured at baseline.

| **Supplemental Table 4.**  **Data on 60-day mortality of patients categorized by their oxygenation-compliance combination on day 21.** | | | | | | | | | | | |
| --- | --- | --- | --- | --- | --- | --- | --- | --- | --- | --- | --- |
|  | **ARDSNet cohort (n=124)** | | | | | **CARDS cohort (n=96)** | | | | | |
|  | MH-LoC (n = 53) | MH-HiC  (n = 25) | SH-HiC (n = 15) | SH-LoC  (n = 31) | p value | MH-LoC (n = 32) | MH-HiC  (n = 36) | SH-HiC (n = 6) | SH-LoC  (n = 22) | p value |  |
| 60-day mortality | 15 (28.3) | 5 (20.0) | 10 (66.7)* | 10 (32.3) | 0.017 | 18 (56.3) | 18 (50.0) | 5 (83.3) | 18 (81.8) | 0.056 |  |

*Abbreviations:* ARDS, acute respiratory distress syndrome; CARDS, COVID-19-related ARDS; MH-LoC, mild-moderate hypoxemia and low compliance; MH-HiC , mild-moderate hypoxemia and high compliance; SH-HiC, severe hypoxemia and high compliance; SH-LoC, severe hypoxemia and low compliance; n, number.

Baseline was day 0 or, if missing, day 1.

* SH-HiC vs MH-LoC, MH-HiC, p<0.05.

**Supplemental Figure 1. Detailed patient flow diagram for both study cohorts.** The “extubated” group consisted of patients who were liberated from mechanical ventilation by day 21; the “deceased” group consisted of patients who died by day 21; and the “ventilated in assisted modes” group consisted of patients who remained on mechanical ventilation but did not have data on compliance, mostly because they were ventilated on assisted or partially assisted modes by day 21 (details on the modes of ventilation are not available for patients in the FACTT ARDSNet trial.). The “ventilated in control modes” group consisted of patients who were on mechanical ventilation and had available data on oxygenation and compliance on day 21.


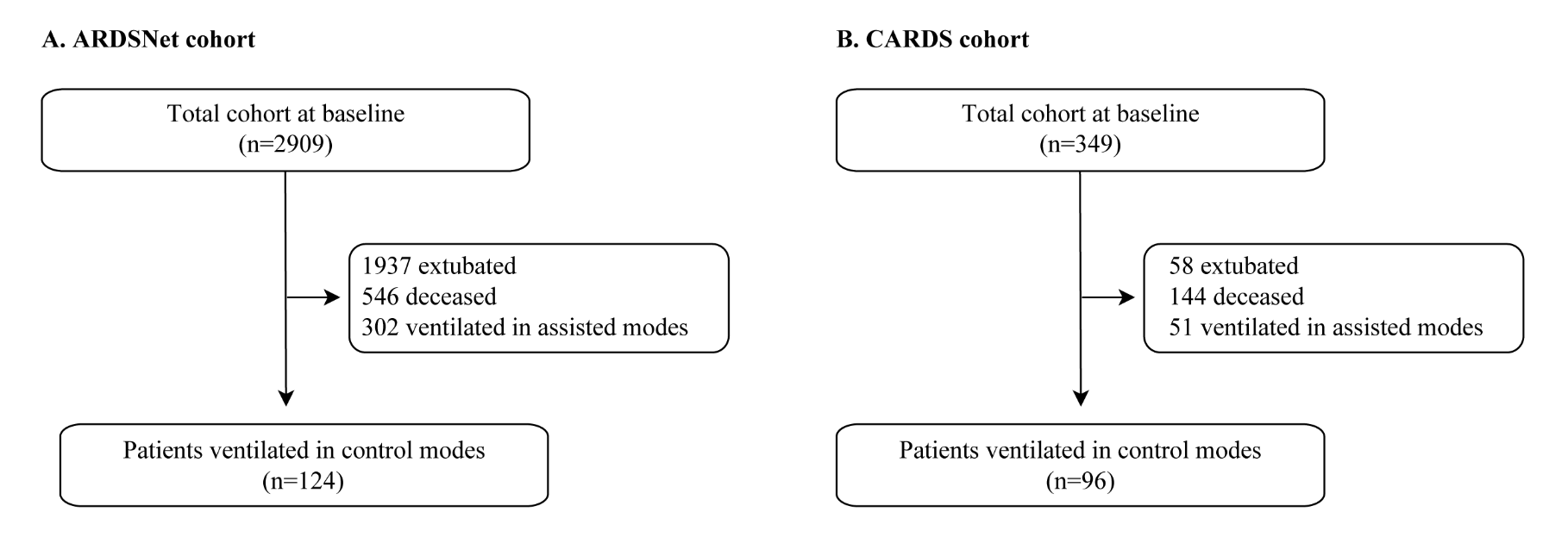


**Supplemental Figure 2. Kaplan-Meier curves of mortality up to day 21 of patients categorized by their oxygenation-compliance combination at baseline.**


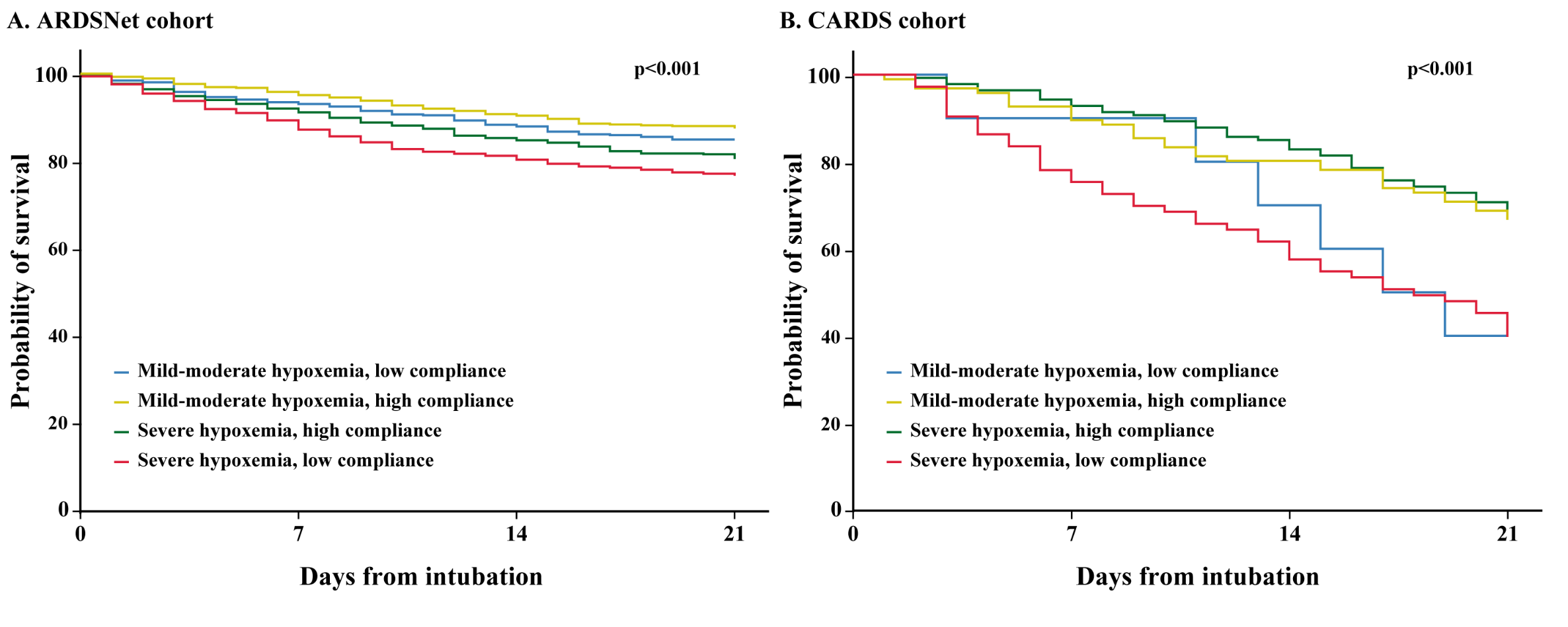

Supplement: Supplementary file 1 — Supplementary Material 1: Supplemental Table 1. Baseline characteristics of patients according to status on day 21. Supplemental Table 2. Baseline characteristics and lung mechanics of the patients with pneumonia as primary risk factor for ARDS. Supplemental Table 3. Outcomes on day 21 of patients categorized by their oxygenation-compliance combination at baseline. Supplemental Table 4. Data on 60-day mortality of patients categorized by their oxygenation-compliance combination on day 21. Supplemental Figure 1. Detailed patient flow diagram for both study cohorts. Supplemental Figure 2. Kaplan-Meier curves of mortality up to day 21 of patients categorized by their oxygenation-compliance combination at baseline [file 12931_2023_2626_MOESM1_ESM.docx]
